# Epicatechin Gallate as Xanthine Oxidase Inhibitor: Inhibitory Kinetics, Binding Characteristics, Synergistic Inhibition, and Action Mechanism

**DOI:** 10.3390/foods10092191

**Published:** 2021-09-15

**Authors:** Miao Zhu, Junhui Pan, Xing Hu, Guowen Zhang

**Affiliations:** State Key Laboratory of Food Science and Technology, Nanchang University, Nanchang 330047, China; 13617912638@163.com (M.Z.); panjunhui@ncu.edu.cn (J.P.); hx0726@ncu.edu.cn (X.H.)

**Keywords:** xanthine oxidase, epicatechin gallate, synergistic inhibition mechanism, superoxide anion, molecular dynamics simulation

## Abstract

Epicatechin gallate (ECG) is one of the main components of catechins and has multiple bioactivities. In this work, the inhibitory ability and molecular mechanism of ECG on XO were investigated systematically. ECG was determined as a mixed xanthine oxidase (XO) inhibitor with an IC_50_ value of 19.33 ± 0.45 μM. The promotion of reduced XO and the inhibition of the formation of uric acid by ECG led to a decrease in O^2−^ radical. The stable ECG–XO complex was formed by hydrogen bonds and van der Waals forces, with the binding constant of the magnitude of 10^4^ L mol^−1^, and ECG influenced the stability of the polypeptide skeleton and resulted in a more compact conformation of XO. Computational simulations further characterized the binding characteristics and revealed that the inhibitory mechanism of ECG on XO was likely that ECG bound to the vicinity of flavin adenine dinucleotide (FAD) and altered the conformation of XO, hindering the entry of substrate and the diffusion of catalytic products. ECG and allopurinol bound to different active sites of XO and exerted a synergistic inhibitory effect through enhancing their binding stability with XO and changing the target amino acid residues of XO. These findings may provide a theoretical basis for the further application of ECG in the fields of food nutrition and functional foods.

## 1. Introduction

Gout is the most common inflammatory arthritis in adults worldwide, which has become the second large metabolic disease after diabetes [1]. According to surveys, more and more patients are plagued by gout every year [2]. The pathogenesis of gout is mainly due to the abnormal metabolism of uric acid. Xanthine and hypoxanthine generate uric acid under the catalysis of xanthine oxidase (XO), and if uric acid is produced excessively or excreted insufficiently, it will accumulate in the body and induce hyperuricemia [3]. Severe hyperuricemia induces gout and is often associated with some complications, such as kidney disease, arthritis, hyperlipidemia, hypertension, diabetes, arteriosclerosis, and coronary heart disease [4]. Currently, foods rich in purine are widely favored by residents, which increases the incidence risk of hyperuricemia and gout. Therefore, the prevention and treatment of gout through daily diet has attracted widespread attention.

XO is a molybdenum-containing flavin enzyme, which is mainly distributed in the liver and kidney and is responsible for the adjustment of purine catabolism. According to the crystal structure analysis of the XO molecule, it contains two identical subunits that can independently play a catalytic role. Each subunit includes three domains: the N-terminal domain with two [2Fe-2S] cluster centers, the flavin adenine dinucleotide (FAD) center, and the C-terminal molybdopterin (Mo-pt) domain, and their roles in the catalytic reaction are to participate in the electron transfer reaction, provide electrons in the redox reaction, and act as the active center of the catalytic reaction [5]. XO can catalyze hypoxanthine to xanthine and then to uric acid and directly catalyze xanthine to uric acid, which is accompanied by the formation of superoxide anion (O_2_^−^) or hydrogen peroxide (H_2_O_2_). Studies have shown that the chronic accumulation of excessive free radicals will damage cell membranes, proteins, lipids, DNA, and other macromolecules, leading to a variety of diseases including stroke, cancer, Alzheimer’s disease, and Parkinsonism [6]. Due to its ability to produce uric acid and reactive oxygen species, hyper-active XO is associated with a variety of human diseases [7,8,9]. As a vital oxidoreductase in the body, the activity of XO directly influences the level of uric acid. Consequently, improving the abnormal metabolism of uric acid by inhibiting the activity of XO has become one of the main ways to relieve gout, which can fundamentally reduce the production of uric acid [10].

Currently, allopurinol and febuxostat are commonly used inhibitors of XO in clinical practice. However, these chemical synthetics often cause adverse reactions such as fever, rash, diarrhea, liver and kidney injury, and thrombocytopenia in clinical use, thus limiting their application [11]. Therefore, it is urgent to discover potential XO inhibitors with high activities and low side effects from natural active components, which have important theoretical and practical values for the prevention or early treatment of hyperuricemia and gout. Dietary interventions with natural XO inhibitors, such as polyphenols, flavonoids, and triterpenes, are safe and effective ways to regulate the level of uric acid in the body [12]. Dong et al. [13] determined the inhibitory activities of five dietary flavonoids on XO and found that these flavonoids significantly inhibited the activity of XO, suggesting that flavonoids can prevent hyperuricemia and its associated complications. The hydrolyzed extracts from anti-hyperuricemic plant parts were reported to act as potent XO inhibitors, and myricetin and apigenin in the extracts played a major role to influence the activity of XO [14]. Free phenolics identified from adlay bran were reported to be predominant XO inhibitors, indicating that adlay is a promising functional food for preventing hyperuricemia and gout [15].

**Figure 1:**
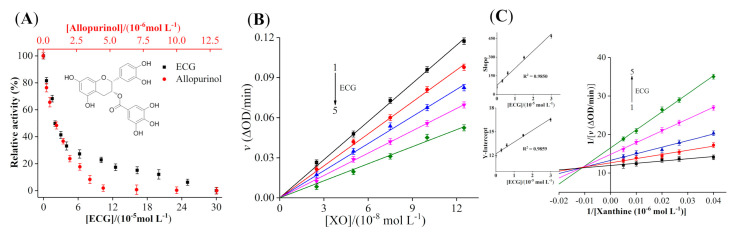
Inhibition analysis. (**A**) The effects of epicatechin gallate (ECG) and allopurinol on the activity of xanthine oxidase (XO) (pH 7.4, T = 25 °C). *c* (XO) = 0.075 μM, *c* (xanthine) = 50.0 μM; (**B**) the plots of ν versus [XO]: *c* (xanthine) = 50.0 μM, *c* (ECG) = 0, 4.0, 8.0, 15.0 and 40.0 μM for curves 1−5, respectively; (**C**) the Lineweaver–Burk plots of XO in the presence of ECG. *c* (XO) = 0.075 μM, *c* (ECG) = 0, 4.0, 8.0, 15.0 and 40.0 μM for curves 1 → 5, respectively. The plots of slope and Y–intercept vs. ECG were inserted to the left.

Epicatechin gallate (ECG, Figure 1A), one of the main components of the catechins, is an active phenolic substance extracted from tea and other natural plants. With the deepening of research on ECG, more pharmacological functions have been discovered, such as antitumor [16], antioxidant [17], anti-inflammatory [18], antidiabetic activities [19], scavenging free radicals, and regulating the metabolism [20]. Due to the multiple functional and active advantages of catechins, they have been widely used in the development of nutraceuticals or functional foods. For instance, green tea catechins can be used in nutraceuticals for body-weight management [21]. Wu et al. [22] reported that catechin incorporated into fermented vinegar may lower the risk of diabetes, which was due to its strong inhibition of the diabetes-related metabolic enzymes. Therefore, catechins can exert their nutritional and health value by inhibiting the activity of some disease-related enzymes. In addition, previous studies have shown that catechins in cocoa leaf extracts were effective XO inhibitors and free-radical scavengers, thus cocoa leaves may be useful for managing gout and preventing oxidative stress [23]. ECG was found to have pharmacological activity of lowering uric acid levels, which was mediated by the inhibition of XO and the effects on related transporters [24]. Currently, many polyphenols with a similar structure to ECG have been determined to be effective XO inhibitors, and their inhibitory mechanisms have been elucidated. For instance, our previous studies found that kaempferol reversibly inhibited XO activity in a competitive manner, and the main inhibitory mechanism may be that kaempferol inserted into the catalytic center of XO, blocked the entrance of substrate, and induced the conformational changes of XO [25]. The insertion of the acyl group of anthocyanins into the hydrophobic region of XO, and the interaction with some key amino acid residues may be the main mechanism of the decline of the catalytic activity of XO [26]. However, current reports were mostly limited to exploring the inhibition capacity of catechins on XO and determining their IC_50_ values, while little has been known about the molecular mechanism of the inhibition of ECG on XO.

High doses of drugs used alone were often accompanied by serious side effects, and the combination of two or more compounds may reduce the dosage and achieve the same or better efficacy [27]. The combination between the extracts from cones of tetraclinis articulata masters and antibiotic or anti-inflammatory agents showed synergism effect, which can reduce their effective antimicrobial and anti-inflammatory dose, thereby relieving their undesirable problems [28]. Mehmood et al. [29] reported that the joint use of stevia residue extract with allopurinol significantly attenuated hyperuricemia, oxidative stress, and inflammation by synergistically inhibiting the activity of XO. Therefore, research on the combined inhibitory effect and mechanism of ECG and allopurinol is also of great practical significance, which may provide a theoretical basis for the research and development of novel anti-hyperuricemia compound medicines with high efficacy and few adverse reactions.

In this study, we used XO as an enzyme target in vitro and innovatively and deeply explored the inhibition kinetics and molecular mechanism of ECG against XO, the binding characteristics between ECG and XO, as well as its combined inhibition and mechanism with allopurinol by using a series of novel research approaches, such as multiple spectroscopic methods including UV–vis absorption, fluorescence and circular dichroism (CD) spectroscopy, fluorescence microscope imaging, along with molecular simulation techniques. In addition, the antioxidant behavior of ECG in the catalytic process of XO was also further investigated. This research is expected to promote the better exertion of the nutritional value ECG and also provide important clues for the development of functional products with rich ECG for the prevention and treatment of hyperuricemia.

## 2. Materials and Methods

### 2.1. Materials

XO from bovine milk (Grade I, 10.87 units mL^−1^) and xanthine (purity ≥ 98%) were supplied by Solarbio Co. (Beijing, China), and their stock solutions with concentrations of 5.00 μM and 0.50 mM were prepared using Tris–HCl buffer (pH 7.4, 0.05 M). ECG (purity ≥ 98%) was purchased from Shanghai Yuanye Biotechnology Co., Ltd. (Shanghai, China) and dissolved in ethanol−water (40%, *v/v*) to prepare the stock solution (5.00 mM). Allopurinol and 2,2-diphenyl-1-picrylhydrazyl (DPPH) from Sigma–Aldrich Chemical. (St. Louis, MO, USA) used dimethyl sulfoxide (DMSO) and absolute ethanol as solvents, respectively. Phenazine methosulfate (PMS), nitrotetrazolium blue chloride (NBT), and triton X−100 were all provided by Sinopharm Chemical Reagent Co., Ltd. (Shanghai, China), while β-NADH (NADH) was obtained from Aladdin Reagent Co. (Shanghai, China), and they were all soluble in ethanol. The proportions of ethanol and DMSO were strictly controlled in all experiments to ensure that they did not influence the results [30]. All other chemical reagents were of analytical purity, and the water used throughout the experiments was ultrapure water.

### 2.2. Instruments and Methods

#### 2.2.1. XO Activity Assay

The activity of XO was determined by the time–kinetics module of a spectrophotometer (UV–2450, Shimadzu, Japan). In the system of Tris–HCl (pH 7.4, 0.05 M) as the solvent, XO (0.075 μM) and different amounts of ECG were mixed and incubated at 37 °C for 30 min. The catalytic reaction was initiated after adding the substrate xanthine (50.0 μM), and then the absorbance of the samples was detected every 10 s at 293 nm. The activity of XO in the absence of ECG was defined as 100%, and the inhibitory capacity of ECG against XO was expressed by the IC_50_ value (the concentration of ECG when the relative activity of XO was 50%). Allopurinol was used as a positive control.

#### 2.2.2. Kinetic Analysis of Enzyme-Catalyzed Reaction

The same determination method of enzyme activity was used to conduct the inhibition kinetics analysis of ECG on XO. In the presence of a series of different concentrations of ECG, the concentration of XO or xanthine was changed to determine the catalytic rate, and then the inhibition type was ascertained by the Lineweaver–Burk plots in double reciprocal form. The mixed inhibition was usually described through the following equations [31]:

(1) $$\frac{1}{v}=\frac{K_{m}}{V_{\max}}\left(1+\frac{\left[I\right]}{K_{i}}\right)\frac{1}{\left[S\right]}+\frac{1}{V_{\max}}(1+\frac{\left[I\right]}{{\alpha K}_{i}})$$

(2) $$\mathrm{Slope}=\frac{K_{m}}{V_{\max}}+\frac{K_{m}\left[I\right]}{V_{\max}K_{i}}$$

(3) $$Y-\text{intercept}=\frac{1}{V_{\max}^{\mathrm{app}}}=\frac{1}{V_{\max}}+\frac{1}{{\alpha K}_{i}V_{\max}}\left[I\right]$$

where [I] and [S] are the concentrations of inhibitor and substrate, respectively. *v* and *V*_max_ represent the catalytic rate and maximum velocity, respectively. *K*_m_, *K*_i,_ and *α* are on behalf of the Michaelis–Menten constant, dissociation constant, and apparent coefficient, respectively.

#### 2.2.3. DPPH Radical Scavenging Activity Assay

The DPPH free radical scavenging activity of ECG was tested according to Aalim et al. with slight modification [32]. DPPH was dissolved in ethanol, and then different concentrations of ECG were added to prepare a series of samples. The mixed solutions were protected from light and reacted for 30 min. The absorbance of the samples at 517 nm was determined by an enzyme-labeled instrument (Varioskan LUX, Thermo Fisher Scientific, Waltham, MA, USA). An equal volume of ethanol was used as a control. The free radical scavenging activity of ECG was assessed by the change of percentage in absorbance.

#### 2.2.4. O_2_^−^ Radical Scavenging Activity Assay

NBT could be reduced to blue formazan by O_2_^−^ radical produced by the PMS–NADH system; thus, this method was used to evaluate the O_2_^−^ radical scavenging activity of ECG [33]. The reaction solutions were prepared by mixing NBT (55.0 μM), NADH (0.27 mM), and different concentrations of ECG, and then 0.2 mL PMS (0.155 mM) was added to initiate the reaction. After the samples were incubated for 15 min, the absorbance at 560 nm was measured. Ethanol was used instead of ECG as the blank group, and the values of decreasing percentage in absorbance were calculated to analyze the scavenging activity of ECG.

#### 2.2.5. The Scavenging Activity Assay of O_2_^−^ Radical Generated by XO

The O_2_^−^ radicals generated during the catalytic process of XO could also be used to assess the antioxidant activity of ECG. The XO (0.70 μM) and different concentrations of ECG were mixed in Tris–HCl buffer and incubated at 37 °C for 30 min. Then, NBT (55.0 μM) and xanthine were added to the samples, respectively, and the absorbance of the mixture at 560 nm was recorded. A small amount of Triton X−100 (0.2%, *v/v*) was added for solubilization and the control group was equal volume ethanol.

#### 2.2.6. Fluorescence Titration Experiments

At three experimental temperatures (25, 31, and 37 °C), XO (1.0 μM) diluted in the Tris–HCl solution was placed in a 1.0 cm cuvette, and then ECG (0–30.0 μM) was added into XO solution successively to conduct the fluorescence titration experiments. After each addition, the fluorescence spectra of the mixed solutions within 300–500 nm were scanned at an excitation wavelength of 280 nm using a fluorescence photometer (F–7000, Hitachi, Japan). The width of the excitation and emission slit was 2.5 nm, respectively. Similarly, the synchronous fluorescence spectra of XO titrated by ECG were measured by setting the difference between excitation and emission wavelength (Δλ_em_) to 15 and 60 nm, respectively. The free XO solution and a mixed sample of XO and ECG were prepared, and then the three-dimensional (3D) fluorescence spectra of the samples were determined; the scanning ranges of both excitation and emission wavelength were 200–600 nm. All fluorescence data were corrected to subtract the internal filtering effect by the following equation [34]:

(4) $$F_{c}{=F}_{m}e^{{(A}_{1}{+A}_{2})/2}$$

where *F*_c_ and *F*_m_ represent the detected and corrected fluorescence intensity values, respectively; *A*_1_ and *A*_2_ are the absorbances of ECG at the excitation and emission wavelengths, respectively.

#### 2.2.7. Measurements of CD Spectra

The CD spectrometer (Bio−Logic MOS 450, Claix, France) was used to determine the CD spectra to analyze the effects of ECG on the secondary structure of XO. The free XO (1.0 μM) and its mixed solutions with different molar ratios ([ECG]/[XO], 0:1, 10:1, 20:1 and 40:1) were prepared, and then the CD spectra of the samples in the range of 190−250 nm were measured in a nitrogen stream. The specific secondary structure contents were analyzed by our previous study [31].

#### 2.2.8. Microstructure Analysis

The free XO solution and the mixture of ECG and XO were prepared and incubated for 1h at room temperature. The appropriate amount of samples was placed on a microscope slide, and then the structural changes of XO induced by ECG were observed through a fluorescent inverted microscope with an excitation light of FBW (IX73, Olympus, Japan).

#### 2.2.9. Molecular Docking

According to the method reported by Dai et al. [35], the molecular docking between ECG and XO was completed by the Discovery Studio 4.5 software (neotrident, Beijing, China). The 3D structure of ECG and the crystal model of XO (PDB ID: 1FIQ) were obtained from the PubChem database and RCSB Protein Data Bank, respectively. The XO model was treated with water removal, hydrogenation, and polarity addition and then defined as a receptor. The molecular docking of the ECG−XO system was performed via the CDOCKER program with the running times and the docking tolerance of 100 and 0.25 Å, respectively [36]. The optimal binding pose of ECG−XO with the lowest binding energy was selected to further analyze the binding sites and main forces.

#### 2.2.10. Molecular Dynamics (MD) Simulation

The binding state of ECG and XO in a relatively real solvent environment was simulated using the GROMACS 5.1 program, and the used force field was AMBER99SB-ILDN. The water was removed from the PDB file of XO, and the ECG topology file was constructed in the Acpype server and then combined to obtain the total topology file. The complex was soaked in a closed dodecahedron water box, and the charges on the surface of the ECG−XO complex were neutralized by adding Na^+^ and Cl^−^. After the energy minimization process, the temperature and pressure of the system were equilibrated at 300 K and 1 bar for 100 ps, and then the MD simulation was performed for 30 ns.

#### 2.2.11. Combined Inhibition Analysis

The combined inhibitory effect of ECG and allopurinol was investigated using the theory of dose-normalized isobologram reported by Chou [37]. The XO activity was determined using the same method described in Section 2.2.1. The dose at which ECG or allopurinol individually reduced the XO activity by 30%, 50%, and 70% was defined as 1. At the constant dose ratios of allopurinol (20%, 40%, 60%, and 80%), the doses of ECG that achieved the same inhibitory effect on XO when used in combination with allopurinol were acquired. The X and Y axes were the dose percentages (*D*_a_ and *D*_b_) of ECG or allopurinol, and their combined effects were denoted in a graph. The joint inhibition effects of two inhibitors were evaluated through the combination index (CI) values as follows:

(5) $$\mathrm{CI}=\frac{D_{a}}{{{(D}_{X})}_{a}}+\frac{D_{b}}{{{(D}_{X})}_{b}}$$

where *D*_x_ denotes the dose of ECG or allopurinol that individually inhibited X% activity of XO. The evaluation theory based on CI values: synergism (CI < 0.9), addition (CI = 0.9−1.1) or antagonism (CI > 1.1).

#### 2.2.12. Statistical Analysis

All experiments were carried out in parallel three times, and the results were expressed as mean ± standard deviation. The data were analyzed by one-way ANOVA using Duncan’s test of OriginPro 9.1, and *p* < 0.05 indicated that there was a significant difference between the results.

## 3. Results

### 3.1. Inhibitory Activity of ECG on XO

Figure 1A showed that both ECG and allopurinol had significant inhibitory effects on XO in a dose-dependent type. The activity of XO was almost completely inhibited when the concentration of ECG reached 300 μM. The IC_50_ values of ECG and allopurinol to inhibit XO were determined as 19.33 ± 0.45 μM and 1.06 ± 0.04 μM, respectively, demonstrating that ECG could significantly inhibit XO activity, but its inhibitory ability was lower than allopurinol, which was different from the IC_50_ value of ECG (0.26 μM) inhibiting XO reported by Yuan et al. [12]; this may be due to the differences in the source of inhibitors and reaction conditions, as IC_50_ value is susceptible to incubation time, concentrations of samples and detection methods. The IC_50_ values of caffeic acid and chlorogenic acid for inhibiting XO activity were 7.16 mM and 6.49 mM, respectively [15]. Our previous studies also found that myricetin and quercetin inhibited the XO activity, with the IC_50_ values of 8.66 μM and 7.23 μM, respectively [38], which were similar to our determined results. The high IC_50_ value of ECG may be due to its bulkier structure, the large steric hindrance of ECG made its binding affinity with XO lower than other polyphenols, hindering the entry of ECG into the active center of XO partly and weakening its ability to inhibit XO activity. Previous studies reported that the functional activities of catechins were enhanced as the number of galloyl moieties was increased [39]; thus, the galloyl group of ECG may be one of the reasons for its high inhibitory potency on XO. Zhao et al. [40] found that polyphenols were excellent inhibitors against XO because their phenolic hydroxyl groups could form hydrogen bonds with the amino acid residues of XO, which enhanced the binding affinity with XO. These reports were similar to our results and may reasonably explain the strong inhibitory effect of ECG on XO.

### 3.2. Inhibition Kinetic Analysis of ECG

In the presence of different amounts of ECG, the effects of gradually increasing the concentration of XO on the catalytic rate were shown in Figure 1B. The curves of *ν* vs. XO with good linearity intersected at the origin, and the slope was inversely proportional to the concentration of ECG. These results indicated that ECG reversibly inhibited the catalytic activity of XO instead of causing complete inactivation of XO. The covalent binding of irreversible inhibitor and enzyme may result in the inactivation of the enzyme due to the formation of a stable complex [30]; thus, the interaction between ECG and XO may be in a non-covalent form.

As shown in Figure 1C, the plots obtained by the double reciprocal mapping method were used to determine the inhibitory type of ECG on XO. All the fitted curves intersected in the second quadrant, and with the increase of concentration of ECG, *V*_max_ and *K*_m_ showed the decreasing and increasing trend, respectively. These phenomena indicated that ECG was a mixed inhibitor and can interact with either free XO or XO−xanthine complex. The plots of slope or intercept vs. the concentration of ECG showed well linearity, manifesting that ECG had only a single or one class of inhibitory site in XO [31]. In addition, the value of *αK*_i_ (82.4 ± 0.70 μM) was greater than *K*_i_ (5.27 ± 0.24 μM), suggesting that ECG had a stronger affinity for free XO than XO−xanthine complex. Sae-Leaw et al. [41] studied the inhibitory effects of several catechins on the melanosis of Pacific white shrimp and found that ECG exhibited a mixed inhibitory effect on polyphenoloxidase. These findings may contribute to further understanding of the inhibitory mechanism of ECG against XO.

### 3.3. The Inhibition of ECG on O_2_^−^ Radical Produced by XO

The enzymatic reaction of XO is a catalytic process with xanthine and O_2_ as substrates and follows the ping-pong mechanism. In other words, XO catalyzes the formation of uric acid from xanthine and turns into intermediates of XO (EI or EII). Then, O_2_ as another substrate is catalyzed by the intermediates to release the O_2_^−^ radical or H_2_O_2_, while the intermediates are restored to the original XO, which completes a cycle [42]. Hence, the pathways for inhibitors to suppress O_2_^−^ radical could be generalized as the follows: (I) XO could be reduced by inhibitors, which makes the intermediate (EII) only catalyze O_2_ to release H_2_O_2_; (II) the production of O_2_^−^ radical is declined by inhibiting the catalytic activity of XO; (III) the O_2_^−^ radical is scavenged by inhibitors.

**Figure 2:**
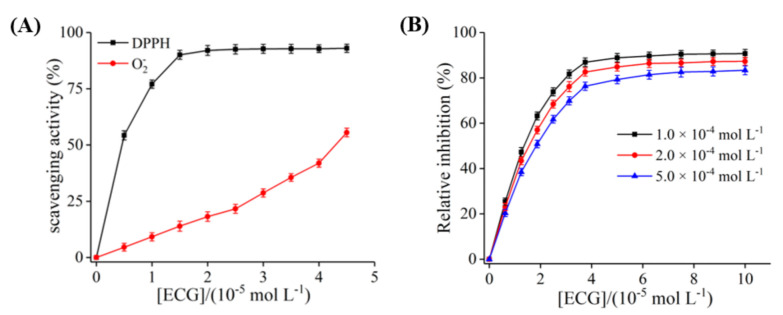
The antioxidant mechanism assay. (**A**) The scavenging effects of ECG on DPPH radical and O^2−^ radical generated from phenazine methosulfate–β-NADH (PMS–NADH) system; (**B**) the inhibitory effects of ECG on the formation of O^2−^ radical catalyzed by XO under different concentrations of xanthine. *c* (XO) = 0.7 μM, *c* (xanthine) = 0.1, 0.2, and 0.5 mM, respectively.

As shown in Figure 2A, ECG scavenged 90% of DPPH radical at a concentration of 15 μM, suggesting that ECG had strong antioxidant potential and the ability to reduce XO molecule, possibly because ECG prevented the penetration of free radicals into the acyl chain regions [31]. With the increase in ECG concentration, the O_2_^−^ radical generated by PMS–NADH system was gradually scavenged, and the scavenging rate was 55.64% when the concentration reached 45.0 μM, indicating that the scavenging ability of ECG on O_2_^−^ radical was weaker than that of DPPH radical (Figure 2A). As shown in Figure 2B, at the same concentration range, the generation of O_2_^−^ radical from the catalytic process of XO could be quickly inhibited by ECG, and the relative inhibition rate eventually remained at 90%. In addition, the dose–response curves showed the same pattern at the different concentrations of xanthine. It was inferred that the decrease in O_2_^−^ radical generated from XO was due to the inhibition of ECG rather than the scavenging effect on O_2_^−^ radical. Moreover, the dose of ECG was 18.45 μM when the relative inhibition rate of the production of O_2_^−^ radical reached 50%, which was close to the IC_50_ value (19.33 μM) of ECG for inhibiting XO to generate uric acid. These results further confirmed that ECG inhibited O_2_^−^ radical by accelerating the formation of the reduced form of XO and inhibiting the production of uric acid. According to the report by Zhao et al. [40], isorhamnetin also played a role in inhibiting O_2_^−^ radical in the XO catalytic system through these two pathways, which was similar to our results.

### 3.4. Fluorescence Quenching and Thermodynamic Analysis

**Figure 3:**
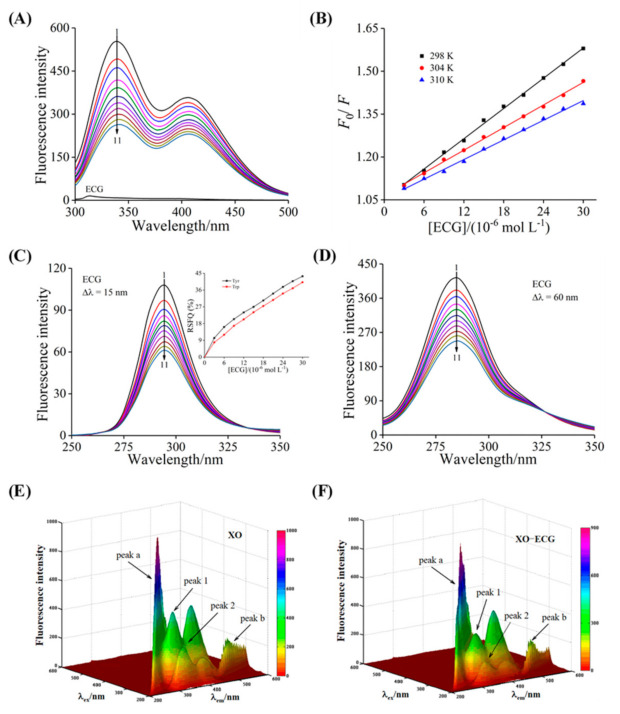
Fluorescence measurements. (**A**) Effects of ECG on the fluorescence emission spectra of XO: *c* (XO) = 1.0 μM, *c* (ECG) = 0, 3.0, 6.0, 9.0, 12.0, 15.0, 18.0, 21.0, 24.0, 27.0, and 30.0 μM for Curves 1→11, respectively; (**B**) the Stern–Volmer plots at 25, 31 and 37 °C. Synchronous fluorescence spectra of XO in the presence of different concentrations of ECG at the Δλ of 15 nm (**C**) and 60 nm (**D**): *c* (XO) = 1.0 μM, *c* (ECG) = 0, 3.0, 6.0, 9.0, 12.0, 15.0, 18.0, 21.0, 24.0, 27.0, and 30.0 μM for Curves 1 → 11, respectively. The insert was RSFQ values of Tyr and Trp. 3D fluorescence spectra of free XO (**E**) and ECG–XO system (**F**): *c* (XO) = 1.0 μM, *c* (ECG) = 15.0 μM.

The changes of fluorescence spectra can reflect the binding characteristics between inhibitor and enzyme [43]. It could be found that the emission spectra of XO showed a strong characteristic peak at 340 nm, while ECG had no characteristic peak under the same conditions (Figure 3A). The intrinsic fluorescence of XO was quenched by the gradually added ECG, while no obvious peak shift was observed, indicating that there was interaction between ECG and XO. The specific quenching mechanism of ECG on XO was determined by the relationship between quenching constant (*K*_SV_) and temperature, and the values of *K*_SV_ and *K*_q_ (the quenching rate constant) were calculated by the well-known Stern–Volmer equation [44].

(6) $$\frac{F_{0}}{F}{=1+K}_{\mathrm{SV}}\left[Q\right]{=1+K}_{q}\tau_{0}\left[Q\right]$$

where *F* and *F*_0_ represent the fluorescence intensity values of XO with and without ECG, respectively; Q is the concentration of ECG; the value of the average fluorescence lifetime of fluorophore for biomacromolecules (*τ*_0_) is 10^−8^ s. Figure 3B showed that the Stern–Volmer plots could be linearly fitted at the three temperatures, indicating that there was one single quenching type. As listed in Table 1, the *K*_SV_ values exhibited a decreasing trend with the increase of temperature, and the *K*_q_ values were higher than the 2.0 × 10^10^ L mol^−1^ s^−1^ (the maximum scattering collision rate constant). Hence, it can be concluded from these results that the fluorescence quenching type of XO by ECG was a single static form, and the main reason was the formation of non-fluorescent ground state complex rather than the dynamic collision [45]. Furthermore, the binding constant (*K*_a_) and the number of binding sites (*n*) were calculated through the following formula:

(7) $$\log\frac{F_{0}-F}{F}{=n\log K}_{a}-n\log\frac{1}{\left[Q_{t}\right]-\frac{{(F}_{0}-F)\left[P_{t}\right]}{F_{0}}}$$

where [*Q*_t_] and [*P*_t_] represent the concentrations of ECG and XO, respectively. The calculated *K*_a_ values were in the magnitude of 10^4^ L mol^−1^ and were inversely proportional to temperature (Table 1), manifesting that the ECG possessed a moderate binding affinity with XO, and the stability of the ECG–XO complex decreased with increasing temperature [46]. The *n* values were close to 1, reflecting that XO had only one class of binding sites for ECG, which was consistent with the conclusions of enzyme kinetics analysis.

The values of thermodynamic parameters of the binding process between ECG and XO, such as entropy change (Δ*S*°), enthalpy change (Δ*H*°), and free energy change (Δ*G*°), were obtained by the van ‘t Hoff equation:

(8) $${\log K}_{a}=-\frac{\Delta H{}^{\circ}}{2.303\mathrm{RT}}+\frac{\Delta S{}^{\circ}}{2.303R}$$

(9) ΔG=ΔH°−TΔS°

where *R* is the gas constant (8.314 J mol^−1^ K^−1^), and *T* is the temperatures of fluorescence titration experiments (25, 31, and 37 °C). As listed in Table 1, Δ*G*° < 0 indicated that the binding of ECG to XO occurred spontaneously. Δ*H*° < 0 and Δ*S*° < 0 demonstrated that hydrogen bonds and van der Waals forces were the main driving forces to stabilize the interaction between ECG and XO [47]. A similar study reported that the formation of the ECG–α-amylase complex was mainly driven by van der Waals and hydrogen bonds [48].

### 3.5. Synchronous Fluorescence Analysis

As the representative fluorescent chromophores, the spectral characteristic peak changes of tyrosine (Tyr) and tryptophan (Trp) residues can reflect the conformational information of XO. The characteristic spectra of Tyr and Trp residues were obtained at Δλ of 15 and 60 nm, respectively. As shown in Figure 3C,D, the addition of ECG caused the fluorescence of Tyr and Trp residues to be gradually quenched with no significant peak shift, suggesting that ECG had almost no effect on the polarity and hydrophobicity of the microenvironment around Tyr and Trp residues [49]. In addition, the ratio of synchronous fluorescence quenching (RSFQ = 1 − *F*/*F*_0_) value of Tyr residue was higher than that of Trp residue, indicating that Tyr residue was closer to the binding site of ECG in XO and thus had a greater contribution to the fluorescence quenching.

### 3.6. The 3D Fluorescence Analysis

The 3D fluorescence spectra can provide more valuable information for the conformational changes of XO induced by ECG. There were four characteristic peaks of XO observed in the wavelength range of 200–600 nm (Figure 3E), peak a (λ_ex_ = λ_em_) and peak b (2λ_ex_ = λ_em_) were the Rayleigh-scattering peak and second-order-scattering peak, respectively, while peak 1 (λ_ex_/λ_em_ = 280/340 nm) and peak 2 (λ_ex_/λ_em_ = 230/335 nm) denoted the characteristic peaks of Trp and Tyr residues and polypeptide backbone structure caused by the intramolecular *π* → *π* * and *n* → *π* * transitions [50]. Figure 3F was the 3D fluorescence spectra of XO after binding with ECG. The intensity of peak a was found to decrease slightly, while peak b remained unchanged, indicating that the formation of the ECG–XO complex weakened the Rayleigh-scattering peak. Moreover, the addition of ECG had the significant quenching effects on peak 1 and peak 2, and the fluorescence intensity values decreased by 34.36% and 47.59%, respectively, while there was no noticeable shift in the peak position, suggesting that ECG basically did not affect the microenvironment of the Trp and Tyr residues but led to changes in the conformational stability of XO. These findings further certified the results obtained from the synchronous fluorescence experiments. Tian et al. [51] measured the 3D fluorescence spectra of the interaction between mononaphthalimide spermidine (MINS) and bovine serum albumin (BSA) and found similar results.

### 3.7. CD Spectra Studies

**Figure 4:**
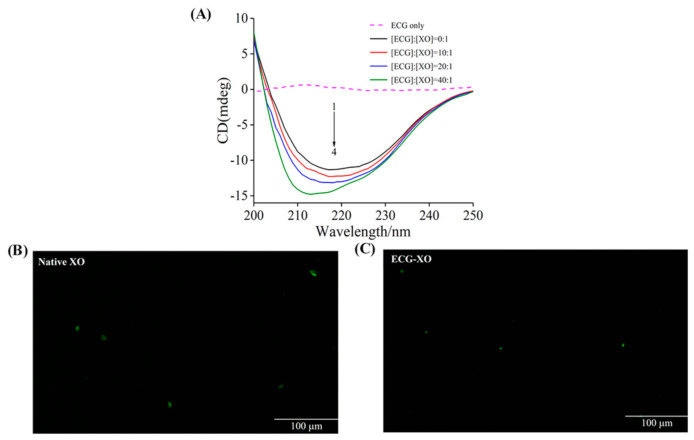
Structural change analysis. (**A**) The CD spectra of XO at different molar ratios of [ECG]:[XO]. *c* (XO) = 1.0 μM, and the molar ratios were 0:1, 10:1, 20:1 and 40:1 for Curves 1 → 4, respectively. Fluorescence microscope imaging of XO (**B**) and ECG–XO (**C**). *c* (XO) = 1.0 μM; *c* (ECG) = 40.0 μM.

The CD spectra of XO in the presence of different molar ratios of ECG to XO were measured to analyze the secondary structure of XO. As shown in Figure 4A, an obvious negative absorption band was observed at around 216 nm for XO, which denoted the characteristic band of β-sheet structure [52]. With the increase of the molar ratio of ECG to XO, the intensity of the CD band increased, and the shape and position of the characteristic peak shifted significantly, which implied that ECG induced the conformational alterations of XO. The specific secondary structure contents calculated using the online program were listed in Table 2. In the presence of ECG, the proportions of α-helix and β-sheet structure of XO raised gradually, while the contents of β-turn and random coil declined. These phenomena might be because the phenolic hydroxyl groups of ECG interacted with XO and formed hydrogen bonds, and such strong force enhanced the rigidity of the XO molecule and caused the structure of XO to become more compact [31]. Therefore, these conformational changes of XO induced by ECG interfered with the entry of substrate into the active center, thus influencing the catalytic function of XO. Wang et al. [53] reported that purpurogallin also inhibited the XO activity through a similar mechanism.

### 3.8. Fluorescence Microscopy Imaging

The effects of ECG on the structure of XO can also be determined by microscopic imaging analysis [54]. As shown in Figure 4B, native XO presented large and bright green plaques, possibly because the chromophoric groups (such as Tyr and Trp residues) in XO caused it to have strong fluorescence under appropriate excitation light. In the presence of ECG, the fluorescence plaques of XO narrowed significantly and turned into small green spots (Figure 4C). The average area of these green bright spots calculated with and without ECG were 172.31 μm^2^ and 49.27 μm^2^, respectively, indicating that the addition of ECG caused a significant decrease in the average area of the bright spots. These phenomena reflected that ECG induced the compact structure of XO, and the protein structure narrowed and the molecule size dwindled, which were consistent with the results of CD spectra.

### 3.9. Molecular Docking

**Figure 5:**
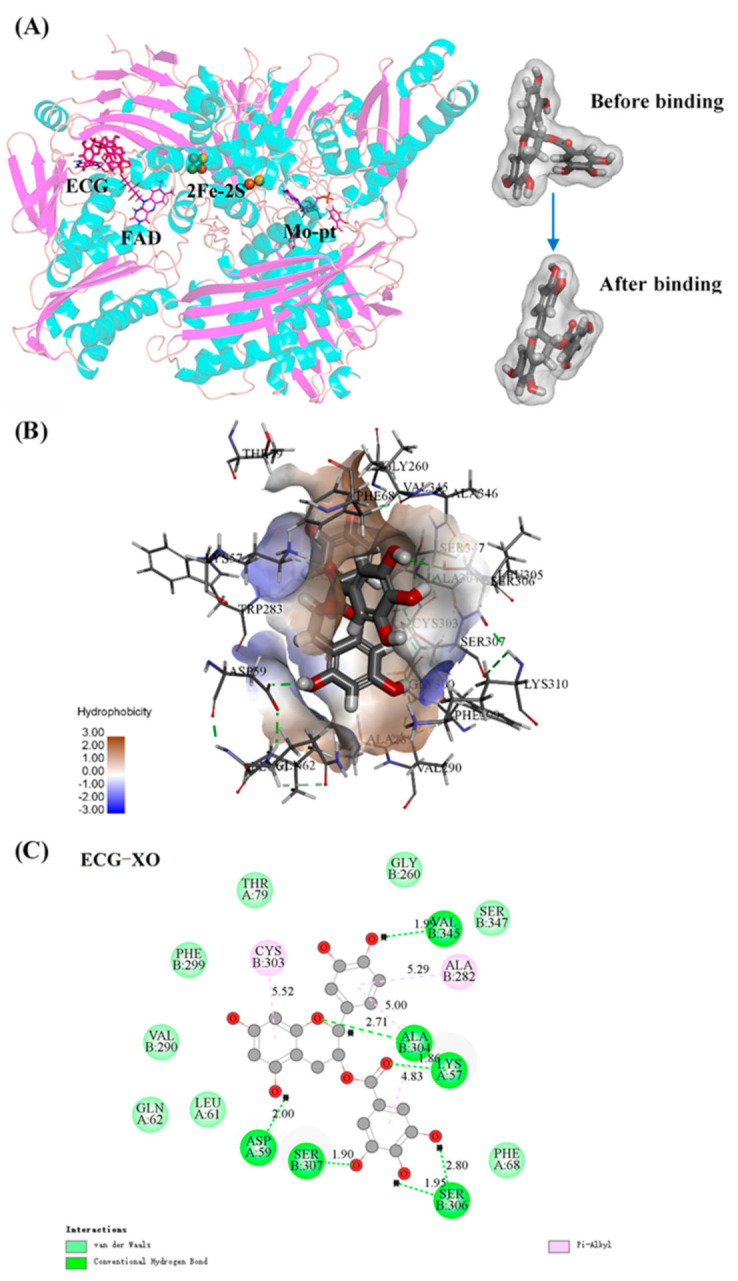
Molecular docking. (**A**) The docking model of ECG–XO complex. The left insets were 3D conformations of ECG before and after binding to XO; (**B**) the hydrophobicity surface of the binding area of ECG in XO; (**C**) the 2D schematic diagram of the binding between ECG and amino acid residues of XO.

Molecular docking can display more visual details of the interaction between ECG and XO. Figure 5A showed the optimal posture of ECG binding to XO with the lowest interaction energy. ECG bound to the vicinity of the FAD site, where the more flexural structure and larger space allowed ECG to insert XO molecule. In addition, the conformation of ECG was found to be significantly distorted after binding to XO (Figure 5A), suggesting that ECG could flexibly adjust its structure to bind with XO [46]. The hydrophobic surface of the 3D docking model and the main amino acid residues involved in the interaction were exhibited in Figure 5B,C; it could be observed that ECG was surrounded by some hydrophobic amino acid residues and formed hydrogen bonds with Lys57, Asp59, Ala304, Ser306, Ser307, Val345 residues, and π–alkyl carbon bonds with Ala282 and Cys303 residues, and van der Waals forces also contributed to strengthening the binding of ECG and XO, which were attributable to the hydroxyl groups and benzene rings of ECG. These findings further confirmed the results of thermodynamic analysis and fluorescence assays. Therefore, in combination with the conclusions of CD spectra, the mechanism by which ECG inhibited the catalytic activity of XO was summarized as that ECG occupied the hydrophobic cavity near the FAD site in XO and interacted with the surrounding amino acid residues to form a more compact rigid conformation, hindering the entry of substrate and the release of catalytic products. Wu et al. [55] also confirmed that ECG covered the active pocket and blocked the entrance of substrate to inhibit the activity of α-glycosidase by the molecular docking analysis, which was similar to our results.

### 3.10. MD Simulation

**Figure 6:**
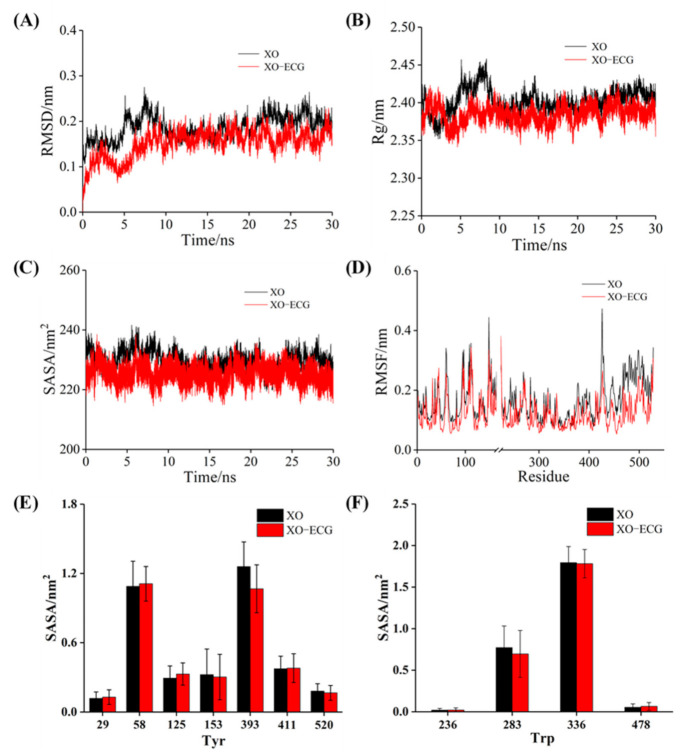
MD simulation of ECG with XO for 30 ns. The RMSD (**A**), Rg (**B**), SASA (**C**), and RMSF (**D**) plots of free XO and ECG–XO complex; the SASA values of Tyr (**E**) and Trp (**F**) residues.

MD simulation was used to characterize the alterations of structure and stability of the binding process of XO and ECG. The root mean square deviation (RMSD) was used to evaluate the stability of the simulated systems. As shown in Figure 6A, the RMSD values of free XO and ECG–XO complex increased and fluctuated significantly during the first 10 ns. The free XO eventually balanced at 22 ns with the RMSD value of 0.21 nm, while the complex system was stable at approximately 0.18 nm after 15 ns. These differences implied that the presence of ECG affected the freedom of the XO structure, and ECG–XO complex had higher stability than the free XO.

The radius of gyration (Rg) is a common index to evaluate the compactness of protein structure [49]. The Rg value of XO was slightly lower than that of free XO during the whole simulation (Figure 6B), indicating that ECG induced XO to form a more compact conformation. The possible reason was that the hydrogen bonds and other forces formed during the binding made the hydrogen bond network structure of XO more stable, which further verified the analysis results of the CD spectra. In addition, it could be seen from Figure 6C that ECG–XO system had a lower solvent accessible surface area (SASA) value than free XO, suggesting that the hydrophobicity of protein increased after interacting with ECG and reflecting a more compact and stable conformation [46], which was consistent with the results of above CD assays.

Root mean square fluctuation (RMSF) can reflect the flexibility of amino acid residues in XO, and the RMSF with greater fluctuation represents higher flexibility [56]. Overall, the RMSF value of the ECG–XO complex was lower and the fluctuation was small (Figure 6D), and the amino acid residues were relatively stable, which denoted that the complex had lower flexibility, which might be because of the increase in the rigid structure of XO induced by ECG hindered the fluctuation of the residues. In contrast, the RMSF values of residues 50–100, 230–350, and 420–530 of XO were significantly different before and after binding with ECG, suggesting that these regions contained more flexible structures and were the locations of amino acid residues involved in the interaction. As illustrated in Figure 6E,F, no significant changes in the SASA values of Tyr and Trp residues were observed before and after binding with the ligand, indicating that ECG basically did not influence the polarity of Tyr and Trp residues, further supporting the conclusions obtained in above spectroscopic experiments.

### 3.11. Joint Inhibitory Effect and Mechanism Analysis

**Figure 7:**
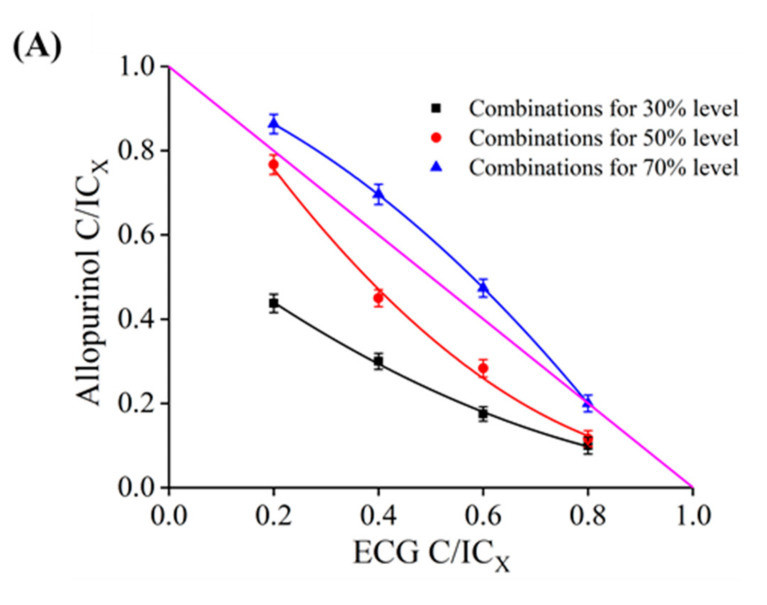

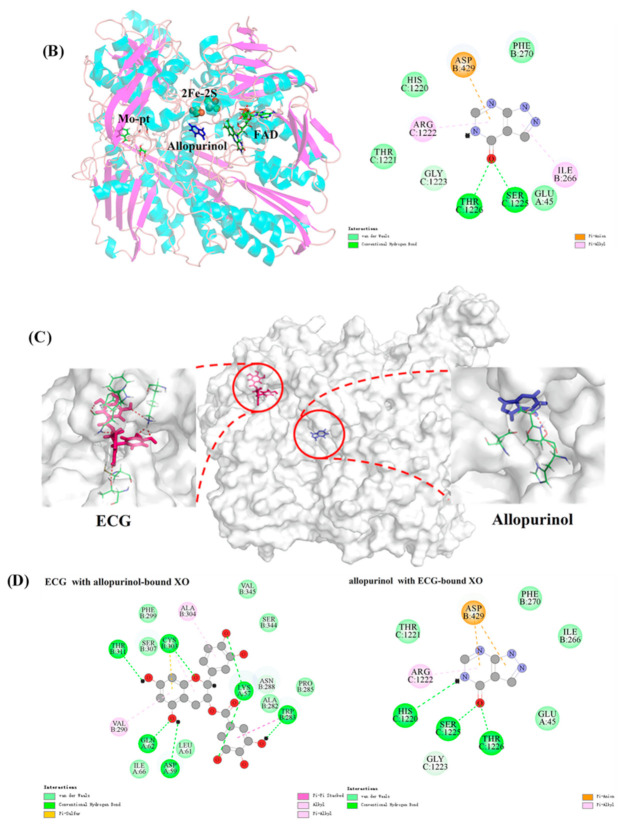
Molecular docking analysis of synergistic inhibition. (**A**) Isoboles of additivity for the combination of ECG and allopurinol at 30%, 50%, and 70% inhibition levels; (**B**) the docking model and 2D schematic diagram of allopurinol–XO complex; (**C**) the 3D interaction pattern of ECG and allopurinol with XO; (**D**) the 2D schematic diagrams of ECG interacting with XO in the presence of allopurinol, and allopurinol interacting with XO in the presence of ECG.

The isoboles of the joint inhibition of ECG and allopurinol on XO were shown in Figure 7A; at the inhibition levels of 30% and 50%, the isoboles were concave and the CI values were less than 0.9, indicating that the combination of ECG and allopurinol exhibited stronger synergistic inhibition on XO. However, the CI values were between 0.98 and 1.09 at the 70% inhibition level, meaning a simple additive effect [31]. Overall, there was a synergistic effect on XO between ECG and allopurinol at low and moderate inhibition levels. These findings suggested that it was feasible to reduce the dose of allopurinol by adding ECG to achieve an equivalent inhibitory effect on XO, which may promote the clinical application of ECG and provide experimental evidence for the pharmacological effects of ECG as a raw material of new compound preparations [57].

Molecular docking of two inhibitors with XO was performed to further explain the mechanism of synergistic effect between ECG and allopurinol. It is well known that the three domains (2Fe-2S, FAD, Mo-pt) in XO molecule are all important active sites in the catalytic reaction, and different types of inhibitors may bind to any site and perturb the binding between substrate and XO, resulting in a decrease in the catalytic activity of XO. Allopurinol, as an analog of xanthine, is a competitive XO inhibitor. As shown in Figure 7B, allopurinol was bound to the cavity near the Mo-Pt center and occupied the binding site of substrate, thus obstructing the normal progress of the catalytic reaction. Allopurinol formed two hydrogen bonds with Thr1226 and Ser1225 residues, and there were also van der Waals forces and hydrophobic interactions between allopurinol and other key amino acid residues. Therefore, the inhibitory mechanism of allopurinol on XO was to compete with xanthine for the binding site. With regard to ECG, the above results found that it inserted into the vicinity of FAD and induced the conformational changes of XO, and blocked the diffusion of the catalytic product (O_2_^−^ radical) out of the FAD region, and disrupted the reaction catalyzed by XO.

The docking results under the joint action of allopurinol and ECG were shown in Figure 7C. These two inhibitors were bound to different active sites in XO. In addition, the amino acid residues involved in the interaction were different between the independent and combined inhibition (Figure 7D). In comparison, there were more hydrophobic forces in the binding of ECG and XO in the presence of allopurinol, and ECG formed a π–sulfur bond with Cys303 residue. Moreover, the molecular docking results revealed that ECG promoted the formation of more hydrogen bonds between allopurinol and key amino acid residues. These enhanced binding forces indicated that ECG and allopurinol strengthened the binding stability and compatibility between each of them and XO. Consequently, we inferred that the synergistic inhibitory mechanism of ECG and allopurinol against XO might be that they bind to different active sites of XO and reduce the activity of XO through different action mechanisms and alter the targeted amino acid residues of XO and strengthen their binding affinity for XO. Ou et al. [58] reported a similar synergistic mechanism in the study of the combined inhibitory effect of kaempferide and galangin on XO.

## 4. Conclusions

The present study demonstrated that ECG was a promising reversible mixed-type XO inhibitor, with the IC_50_ value of 19.33 ± 0.45 μM. The inhibition of ECG on O_2_^−^ radical was achieved by reducing the XO molecule and suppressing the formation of uric acid. ECG quenched the fluorescence of XO in a static manner and was bound to XO by hydrogen bonds and van der Waals forces. The presence of ECG influenced the stability of the polypeptide skeleton of XO but had little effect on the polarity of Tyr and Trp residues. ECG induced an increase in the rigid structure content of XO and resulted in its structural compactness. Molecular docking indicated that ECG was inserted near the FAD site, which limited the flexible movement of the substrate and hindered the diffusion of catalytic products, and ultimately, inhibited the catalytic activity of XO. MD simulation exhibited that the formation of ECG–XO complex reduced the molecular flexibility and induced a more compact conformation. Furthermore, ECG and allopurinol interacted with amino acid residues of different active sites in XO and achieved a synergistic inhibitory effect by enhancing their binding affinity for XO and changing the target residues of XO. This research revealed the action mechanism of ECG as a natural XO inhibitor, which may contribute to the further development of ECG-rich functional foods with the efficacy in lowering uric acid and preventing oxidative damage, although more studies in vivo should be carried out in the future.

## Figures and Tables

**Table 1:** The values of quenching constant (*K*_sv_), binding constant (*K*_a_), number of binding sites (*n*) and thermodynamic parameters of the interaction between ECG and XO at 25, 31, and 37 °C.

*T* (K)	*K*_sv_ (×10^4^ L mol^−1^)	*R* ^a^	*K*_a_ (×10^4^ L mol^−1^)	*R* ^b^	*n*	Δ*H*° (kJ mol^−1^)	Δ*G*° (kJ mol^−1^)	Δ*S*° (J mol^−1^ K^−1^)
298	1.76 ± 0.02	0.9982	1.82 ± 0.05	0.9934	0.91 ± 0.01	−25.05 ± 0.20	−24.32 ± 0.40	−2.44 ± 0.07
304	1.32 ± 0.03	0.9984	1.51 ± 0.02	0.9928	0.95 ± 0.02		−24.31 ± 0.35	
310	1.15 ± 0.01	0.9964	1.23 ± 0.04	0.9985	0.87 ± 0.02		−24.29 ± 0.28	

**Table 2:** The secondary structure contents of XO in the presence of different molar ratios of ECG.

Molar Ratio [ECG]:[XO]	α-Helix (%)	β-Sheet (%)	β-Turn (%)	Random Coil (%)
0:1	8.41 ± 0.21	41.59 ± 0.36	22.27 ± 0.36	27.73 ± 0.36
10:1	10.83 ± 0.51	42.51 ± 0.41	21.65 ± 0.94	25.12 ± 0.69
20:1	10.96 ± 0.62	43.41 ± 0.74	21.59 ± 0.13	24.03 ± 0.31
40:1	11.83 ± 0.93	45.02 ± 0.22	21.39 ± 0.25	22.51 ± 0.24
